# Comparative efficacy of ultrasonic, diode laser, and manual irrigant activation around an apically separated instrument: an in vitro study

**DOI:** 10.1186/s12903-026-09982-0

**Published:** 2026-10-21

**Authors:** Kareem Ayman Asfor, Nihal Adel Leheta, Sybel Mokhtar Moussa

**Affiliations:** Division of Endodontics, Conservative Dentistry Department, Faculty of Dentistry, Alexandria University, Azarita, Alexandria 21521 Egypt

**Keywords:** Ultrasonic, Diode laser, Irrigant activation, Separated instrument

## Abstract

**Background:**

The fracture of a nickel-titanium instrument in the apical third of a root canal presents a significant clinical challenge, as it creates a compacted barrier of dentinal debris and smear layer that is shielded by a vapor lock, severely limiting irrigant efficacy. This study aimed to compare the efficacy of Ultrasonic Activation (UAI), Diode Laser-Activated Irrigation (LAI), and Manual Dynamic Agitation (MDA) in overcoming this barrier by evaluating both irrigant penetration and direct debris removal.

**Methods:**

Ninety human mandibular first molars with standardized, non-bypassable apically fractured instruments were divided into two parts. Part 1 (*n* = 45) assessed irrigant penetration using Confocal Laser Scanning Microscopy (CLSM) after activation of Rhodamine B dye. Part 2 (*n* = 45) evaluated debris and smear layer removal using Scanning Electron Microscopy (SEM) and the Hülsmann scoring system. In both parts, teeth were randomly assigned to one of three final irrigation activation protocols (*n* = 15 each): MDA (as a control group), UAI, or LAI (970 nm diode laser at 2.0 W).

**Results:**

CLSM analysis revealed that UAI achieved a significantly greater penetration area in the apical section compared to LAI (*p* = 0.04). Crucially, UAI was the only technique that showed no significant difference in penetration area between the obstructed apical section and the unobstructed control section (*p* = 0.10). SEM evaluation demonstrated that both UAI and LAI were significantly more effective at removing debris and smear layer than MDA (*p* < 0.05), with no statistically significant difference between UAI and LAI (*p* ≥ 0.76).

**Conclusions:**

Within the limitations of this in-vitro model, ultrasonic activation produced greater apical penetration area than diode laser activation, while ultrasonic and diode laser activation produced significantly better debris and smear-layer removal than manual dynamic agitation. No statistical difference was observed between ultrasonic and diode laser activation for SEM-based cleaning scores. Further studies incorporating thermal safety assessment and biofilm models are required before clinical recommendations can be made.

## Introduction

 A successful root canal treatment necessitates thorough chemomechanical debridement of the root canal system [1]. This effective chemomechanical debridement requires synergistic instrumentation and irrigation. However, the fracture of a nickel-titanium (NiTi) instrument in the apical third presents a formidable obstacle, compacting dentinal debris and smear layer apical to the fragment. The clinical challenge extends beyond the mere physical presence of the metal; the fracture event often compacts dentinal debris and pulp tissue into the intricate apical anatomy, forming a dense, primary barrier to irrigant diffusion [2]. This packed substrate is further shielded by the phenomenon of “vapor lock,” wherein entrapped air bubbles prevent fluid contact with the dentinal walls, reducing the efficacy of sodium hypochlorite (NaOCl) by up to 47% [3, 4]. Consequently, any effective irrigant activation strategy in this scenario must achieve two goals: first, penetrate and disrupt this compacted debris barrier, and second, overcome the vapor lock to enable adequate debridement.

Retrieval attempts of separated instruments, while sometimes successful, carry a significant risk of iatrogenic errors, including perforations, dentinal microcracks, and a substantial reduction in root strength [3]. Recognizing these risks, expert consensus (2025) guidelines prioritize leaving the fragment in situ depending on irrigation activation strategies as a conservative alternative when retrieval is deemed too hazardous or the fragment is non-bypassable [3]. Conventional positive-pressure irrigation is incapable of overcoming these combined barriers [5]. Therefore, the use of adjunctive activation techniques to potentiate irrigant flow beyond the obstruction is essential [5].

Several irrigant activation methods offer distinct hydrodynamic mechanisms to address this challenge. Manual Dynamic Agitation (MDA) utilizes a master gutta-percha cone with a pumping motion to generate transient fluid oscillations and shear stresses, physically displacing vapor-locked bubbles and debris [6]. In contrast, Ultrasonic Activation (UAI), also referred to in the literature as Passive Ultrasonic Irrigation (PUI), operates through powerful acoustic streaming and cavitation; the implosion of cavitation bubbles produces microjets capable of dislodging both trapped air and debris from canal complexities [7]. A third modality, Laser-Activated Irrigation (LAI), employs photonic energy. Diode lasers, with wavelengths such as 970 nm, are absorbed by water, generating photothermal convection currents within the irrigant that can disrupt stagnant areas without contacting the obstruction [8], While UAI achieves 97% irrigant exchange within 0.5 mm of fractures, diode lasers remain untested in this context [9].

However, a critical gap exists in the evaluation of these activation techniques. While studies often quantify irrigant penetration as a proxy for efficacy [10, 11], this metric does not directly measure the removal of the compacted dentinal debris that constitutes the primary clinical barrier. A technique may facilitate dye diffusion through micro-gaps without effectively dislodging the obstruction, thereby overestimating its true cleaning potential. Therefore, a method that directly quantifies debris removal is essential to complement penetration data and provide a clinically translatable assessment of efficacy.

A comprehensive evaluation of irrigation efficacy in this context requires a multi-faceted approach. While the analysis of irrigant penetration patterns using confocal laser scanning microscopy (CLSM) provides valuable, quantifiable data on fluid dynamics, a complete assessment must also directly measure the ultimate clinical goal: the physical removal of the compacted debris barrier. To this end, the present study employs a dual-method analytical strategy, combining CLSM with direct quantitative evaluation of debris removal using scanning electron microscopy (SEM). This approach is designed to provide a more complete and clinically relevant understanding of each technique’s cleaning efficacy.

Therefore, the study aimed to compare the efficacy of Ultrasonic Activation (UAI), Diode Laser-Activated Irrigation (LAI at 2.0 W), and Manual Dynamic Agitation (MDA) in overcoming the barrier of an apically fractured NiTi instrument by simultaneously evaluating irrigant penetration depth and area using CLSM and directly quantifying debris and smear layer removal using SEM. The null hypothesis was that no significant differences exist among the three activation techniques in terms of irrigant penetration parameters (depth, area, arc length) or in the removal of debris and smear layer.

## Materials & methods

### Sample size calculation

Sample size was calculated based on the SEM-assessed debris removal score as the outcome used for sample-size estimation, with a significance level of 5% and a statistical power of 80%. Based on Elkhodary and Morsy [12], mean (SD) debris scores for diode laser, passive ultrasonic, and conventional irrigation were 1.58 (0.51), 1.92 (0.79), and 2.75 (1.06), respectively, at the coronal level; 1.25 (0.45), 1.67 (0.78), and 3.08 (0.90) at the middle level; and 2.08 (0.90), 2.50 (0.80), and 3.33 (0.65) at the apical level. Using the most conservative of these estimates, corresponding to a Cohen’s effect size of approximately f = 0.60, a minimum sample size of 10 teeth per group was required for a three-group comparison at α = 0.05 and 80% power. The calculated sample size was increased to 15 teeth per group to increase precision and compensate for potential specimen loss or laboratory processing errors. This sample size was used separately for the CLSM and SEM experiments, resulting in 45 teeth for CLSM and 45 teeth for SEM and a total final sample of 90 teeth. Sample-size calculation was performed using G*Power version 3.1.9.7.

### Sample selection and preparation

A total of 112 human mandibular first molars were selected that was extracted due to periodontal problems with intact roots and mature apices. Teeth with previous root canal treatment, cracks, fractures, internal resorption, caries, calcification, or developmental anomalies were excluded. The mesial roots exhibited moderate curvature (< 25°) measured according to Schneider’s method [13].

All teeth were debrided, sterilized via autoclaving (121 °C, 15 psi, 20 min), and stored in 0.1% thymol solution. A conventional access cavity was prepared. Teeth were occlusally reduced to a standardized length of 19 mm. Canal patency was established with a #10 K-file, and working length (WL) was set 1 mm short of the anatomical apex.

### Chemomechanical preparation and fracture induction

The mesiobuccal canals were instrumented to size 25/0.04 using PlexV files (ORODEKA, China) in continuous rotation (500 RPM, 2.5 N · cm torque). Irrigation was performed with 3 mL of 2.5% NaOCl between each file using a 30-gauge side-vented needle (Endotop^®^, Cerkamed, Poland) placed 2 mm short of WL.

A size 30/0.04 Plex V file was notched 3 mm from its tip using a diamond disk. The weakened file was inserted into the canal and operated at 1000 RPM under aggressive apical pressure until fracture occurred. Digital radiography was used to confirm that the fractured segment was positioned within 1 mm of the radiographic apex; otherwise, the sample was excluded. Canals where the fragment was bypassable with a #10 K-file were also excluded. Ultimately, 90 teeth out of 112 met all criteria and were randomized using a computer-generated random number table into two halves, as the study was conducted in two parts: -.

- Part 1 (CLSM): 45 teeth (*n* = 15 per activation technique) for irrigant penetration analysis.
- Part 2 (SEM): 45 teeth (*n* = 15 per activation technique) for debris and smear layer analysis.

### Final irrigation and activation protocols for CLSM samples

All groups received a final irrigation sequence of 3 mL of 17% EDTA followed by 3 mL of 2.5% NaOCl. Each 1 mL aliquot was introduced into the canal and activated for 60 s according to group assignment:

- Group (MDA) as a control group: Activation was performed using a size 45 gutta-percha cone with a pumping motion (approx. 100 strokes/60 sec).
- Group (UAI): Activation was performed using an E98 tip “ultrasonic endodontic tip with a #25 diameter and a 2% taper” (Endo 3^®^, Woodpecker, China) at power setting E2 (moderate power), placed 1 mm coronal to the fractured file.
- Group (LAI): Activation was performed using a 970 nm diode laser (Sirolase Blue^®^) with a 200 μm intracanal fiber tip (Easytip^®^). The fiber tip was passively advanced to a position 1 mm coronal to the fractured instrument under microscopic guidance (×16). Activation was performed at 2.0 W power in continuous wave mode for 3 cycles of 20 s each, with a 10-second interval between cycles.

Following irrigant activation, 1 mL of 0.1% Rhodamine B dye in NaOCl solution was introduced and activated for an additional 60 s using the respective group technique. All canals were immediately rinsed with 5 mL of distilled water and soaked in distilled water for 24 h.

### CLSM sample processing and analysis

**Fig. 1:**
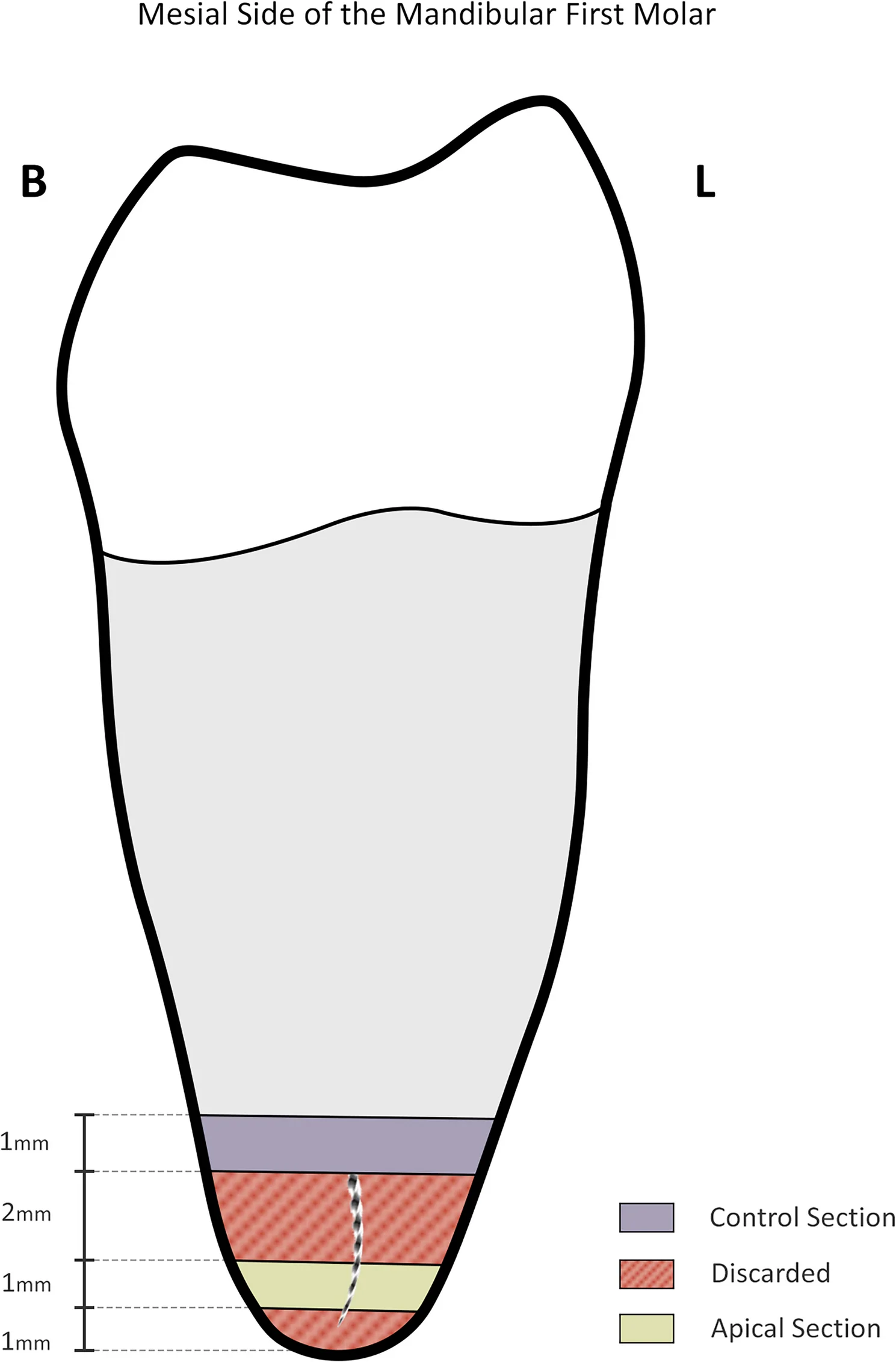
Schematic representation of root sectioning. A diagram illustrating the two sectioning levels performed on each root. The Apical section (red shaded line) was obtained 1 mm coronal to the anatomical apex. The Control section (purple shaded) was obtained 5 mm coronal to the apex, in an unobstructed region of the canal. The fractured file segment is depicted in the apical third of the canal

Following the irrigation and activation procedures, all roots were horizontally sectioned at two levels using a diamond disc (Isomet 4000, Buehler, USA) under water cooling (Fig. 1):

1. Apical Section: A 1-mm-thick section was obtained 1 mm coronal to the anatomical apex. This level corresponded to the region immediately adjacent to the fractured fragment, as the fragment tip was located within 1 mm of the radiographic apex. The fragment remained in situ during sectioning, and this section was used to assess irrigant penetration in the dentinal tubules at the level of the obstruction.
2. Control Section: A 1-mm-thick section was obtained 5 mm coronal to the anatomic apex (a level confirmed to be coronal to the obstruction in all samples). This section served as an internal control to compare irrigant penetration in an unobstructed region of the same root. “.

All sections were imaged using a CLSM (LSM 980, Carl Zeiss, Germany). Rhodamine B was excited at 561 nm, and emission was captured between 570 and 620 nm.

**Fig. 2:**
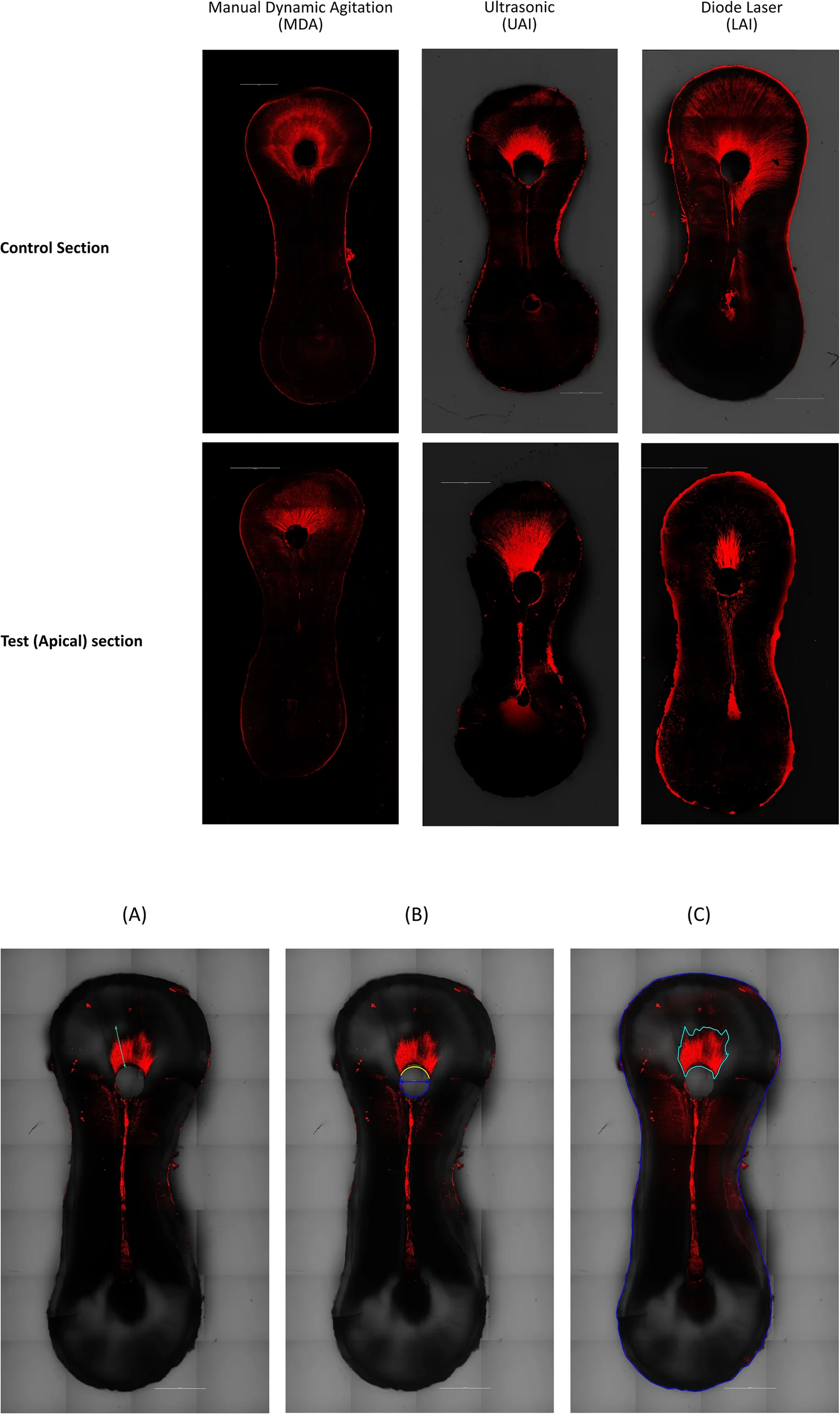
Confocal laser scanning microscopy (CLSM) images & measure parametersRepresentative CLSM images comparing irrigant penetration in Control vs. Apical sections. Paired confocal laser scanning microscopy (CLSM) images of root dentin cross-sections from the same tooth, demonstrating Rhodamine B dye penetration in the unobstructed Control region (5 mm coronal to apex) and the obstructed Apical region (1 mm coronal to apex, beyond the fractured instrument). **A** Maximum Penetration Depth (MPD): The linear distance (μm) from the canal wall (green arrow) to the deepest point of dye penetration. **B** Penetration (Arc Length) Percentage: The proportion of the canal circumference (yellow line) exhibiting dye penetration to the whole canal circumference (blue line). **C** Area of Penetration Percentage: The total area of dye penetration (identified with light blue) relative to the total area of the root section (Identified with Dark blue)

Three parameters were analyzed by two blinded investigators using ZEN 3.2 software (blue edition, Carl Zeiss) for both the apical and control sections:

1. Maximum Penetration Depth (MPD): The linear distance (µm) from the canal wall to the deepest single point of dye penetration within dentinal tubules [14]. (Fig. 2.A)
2. Penetration (Arc Length) Percentage (%): The proportion of the root canal circumference exhibiting dye penetration, expressed as a percentage [15]. (Fig. 2.B)
3. Area of Penetration (%): The total area of dye penetration (µm²) divided by the total area of the root section, expressed as a percentage [16]. (Fig. 2.C)

### SEM samples processing & analysis

The roots were prepared for SEM evaluation by grooving longitudinal ditches on the buccal and lingual surfaces using a diamond disc under water cooling, without penetrating the root canal space. The roots were then carefully split into two halves using a bi-beveled chisel. The fractured instrument segment was removed carefully from the canal to allow for unobstructed visualization of the canal walls. The specimens were soaked in fixative solution 4:1 ratio of 2% glutaraldehyde, 2.5% formaldehyde at PH 7.5 for 24 h. The specimens were washed by PBS solution 4 times, 20 min each, then dehydrated using gradient concentrations of ethanol (30% to 90%) 5 min each, then dehydrated in 100% ethanol 2 times, 10 min each. The specimens were dried using a desiccator for half an hour. The specimens were mounted on an aluminum stub using carbon tape and sputter-coated in vacuum with a gold-palladium layer of thickness 20 nm. After that, the specimen was examined using SEM.

The samples were examined under a scanning electron microscope (JSM-5510LV, JEOL, Ltd, Japan). Imaging was focused on the root canal wall in the apical third, specifically at a level 2 mm coronal to the anatomical apex, to assess the area adjacent to the site of the fractured instrument.

The obtained micrographs were evaluated by two calibrated, blinded investigators for the presence of smear layer and debris separately. Representative images were taken at different magnifications to optimally visualize each parameter: lower magnification (500x) was used for the overall assessment of debris distribution [17], while higher magnification (1500x) was used to evaluate the smear layer and the patency of dentinal tubules [17], according to the Hülsmann scoring system [18], as follows:

Smear layer scores:

- Score 1: No smear layer; dentinal tubules open.
- Score 2: Small amount of smear layer; some dentinal tubules open.
- Score 3: Homogenous smear layer covering the root canal wall; only a few dentinal tubules open.
- Score 4: Complete root canal wall covered by a homogenous smear layer; no open dentinal tubules.
- Score 5: Heavy, nonhomogeneous smear layer covering the complete root canal wall.

Debris scores:

- Score 1: Clean root canal wall; only a few small debris particles.
- Score 2: Few small agglomerations of debris.
- Score 3: Many agglomerations of debris covering less than 50% of the root canal wall.
- Score 4: More than 50% of the root canal wall covered by debris.
- Score 5: Complete or nearly complete root canal wall covered by debris.

### Reliability analysis

Inter-examiner agreement was assessed using Cohen’s weighted kappa (κ) for the ordinal Hülsmann scores. The weighted kappa values were 0.82 for smear layer scores and 0.79 for debris scores, indicating almost perfect and substantial agreement, respectively (Landis & Koch, 1977) [19]. In cases where scores differed by more than one point, a third calibrated investigator was consulted to reach a consensus.

### Statistical analysis

All statistical analyses were performed using IBM SPSS Statistics for Windows (Version 26.0). The normality of data distribution for all outcome measures was assessed using descriptive statistics, Q-Q plots, histograms, and the Shapiro-Wilk test. As the data violated the assumptions of normality, non-parametric tests were employed.

Descriptive statistics were calculated and presented as means ± standard deviation (SD) and medians with interquartile ranges (IQR). For comparisons of the three activation techniques within the same section, the Kruskal-Wallis H test was used. When a significant difference was detected, post-hoc pairwise comparisons were conducted with a Bonferroni adjustment to control for Type I error.

The Wilcoxon signed-rank test was used to compare the penetration parameters between the Apical and Control sections within each activation group. A p-value of less than 0.05 was considered statistically significant for all tests.

## Results

### Irrigant penetration

The results of the irrigant penetration analysis are summarized in Table 1. The data for all outcomes followed a non-normal distribution.

**Table 1:** Comparison of penetration area percentage, Max. penetration depth and arc length percentage between the study groups

			Manual Dynamic Agitation	Ultrasonic	Diode Laser	*P* value 1
Penetration Area %	Test	Mean (SD)	10.98 (9.76) ab	13.91 (7.87) a	5.52 (3.31) b	0.04*
		Median (IQR)	6.44 (4.98, 13.53)	16.37 (5.05, 19.13)	4.76 (3.91, 7.20)	
	Control	Mean (SD)	22.34 (13.89)	17.80 (9.66)	12.40 (8.12)	0.17
		Median (IQR)	21.25 (12.45, 26.21)	20.27 (8.24, 25.54)	11.43 (4.90, 16.32)	
	**P value 2**		0.003*	**0.10**	0.008*	
Maximum Penetration depth	Test	Mean (SD)	705.45 (233.04)	630.29 (315.24)	490.07 (242.16)	0.14
		Median (IQR)	687.27 (595.65, 863.39)	630.29 (331.12, 934.41)	510.74 (308.13, 582.98)	
	Control	Mean (SD)	1225.73 (210.76)	1129.72 (439.49)	1113.63 (713.15)	0.85
		Median (IQR)	1190.22 (1085.60, 1413.37)	1129.72 (666.57, 1410.83)	1180.09 (448.16, 1447.86)	
	**P value 2**		0.003*	0.004*	0.003*	
Penetration (Arc length) %	Test	Mean (SD)	25.08 (14.39)	23.74 (8.34)	24.52 (6.34)	0.95
		Median (IQR)	23.84 (11.15, 34.94)	23.74 (18.71, 28.44)	23.46 (20.08, 25.48)	
	Control	Mean (SD)	63.38 (24.48) a	45.45 (18.71) ab	42.45 (14.57) b	0.04*
		Median (IQR)	63.38 (40.43, 85.28)	45.56 (31.19, 63.10)	42.12 (28.26, 47.10)	
	**P value 2**		0.003*	0.003*	0.008*	

*P* value 1: Comparisons between the three activation methods using Kruskal Wallis H test

*P* value 2: Comparisons between the test and control within each group using Wilcoxon signed ranks test

*SD* Standard Deviation, *IQR* Interquartile Range

*Statistically significant at *p*-value <0.05

a-b: different letters denote significant differences between groups using Bonferroni correction

### Penetration area %

**Fig. 3:**
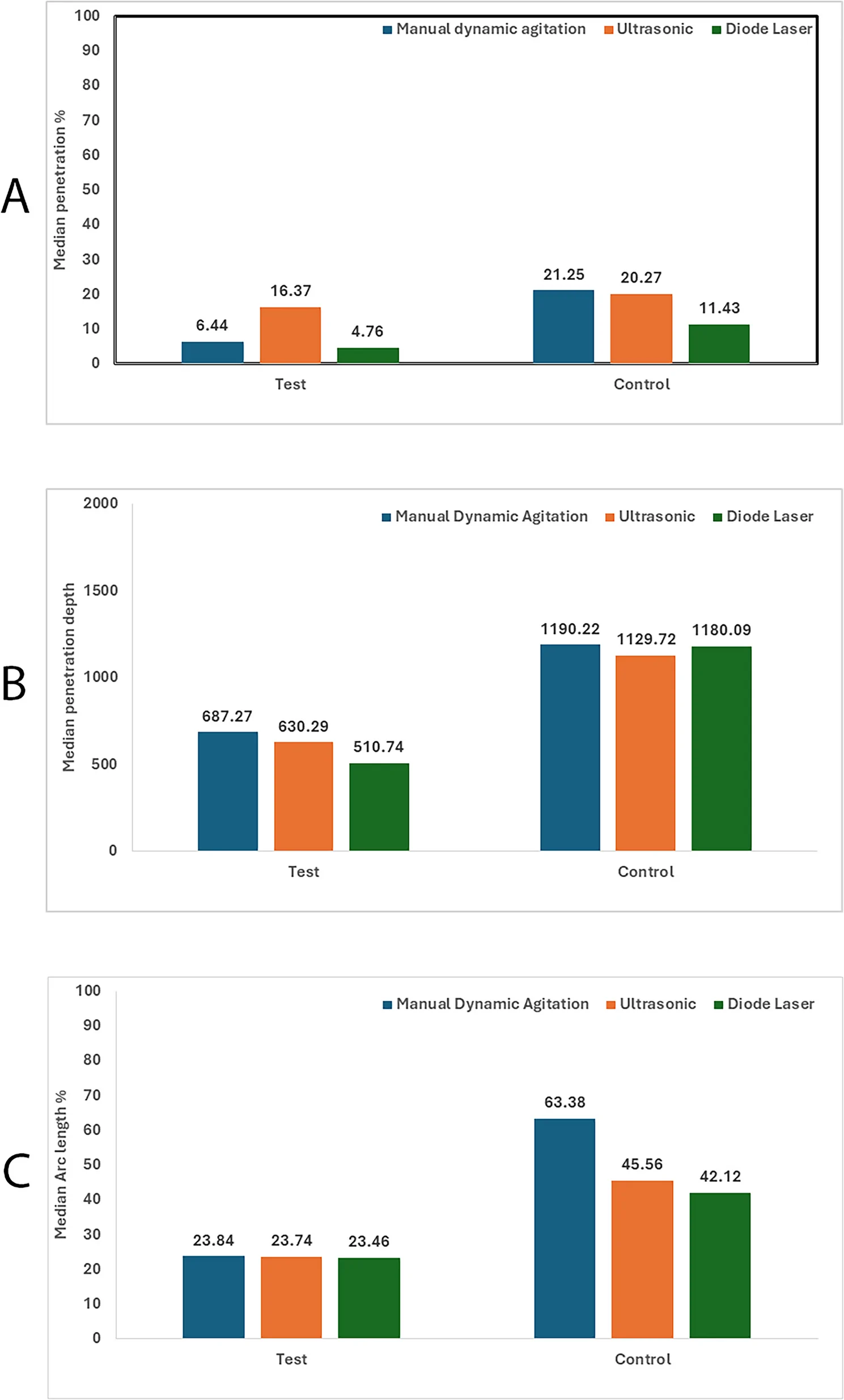
Graphical representation of irrigant penetration results. Bar charts (medians with interquartile ranges) comparing the three irrigation activation techniques—Manual Dynamic Agitation (MDA), Ultrasonic Activation (UAI), and Diode Laser Activation (LAI)—for the Apical (test) and Control sections. **A** Penetration Area (%): UAI achieved a significantly greater penetration area in the Apical section compared to LAI (*p* = 0.04). Notably, UAI’s performance in the Apical section was not significantly different from its Control section (*p* = 0.10). **B** Maximum Penetration Depth (μm): No significant differences were found between the groups in the Apical section (*p* = 0.14). All techniques showed significantly reduced penetration depths in the Apical section compared to their controls (*p* ≤ 0.008 for MDA and LAI; *p* = 0.004 for UAI). **C** Penetration (Arc Length) Percentage (%): The proportion of the canal circumference penetrated was comparable between all techniques in the Apical section (*p* = 0.95). In the Control section, MDA achieved a significantly greater arc length than LAI (*p* = 0.04)

A significant difference was found in the Penetration Area % in the apical section among the three activation techniques (*p* = 0.04). Post-hoc analysis revealed that UAI (Median = 16.37%, IQR = 5.05–19.13) achieved a significantly greater penetration area than LAI (Median = 4.76%, IQR = 3.91–7.20; *p* = 0.04) (Table 2). The performance of MDA (Median = 6.44%, IQR = 4.98–13.53) was not statistically different from either UAI or LAI (*p* > 0.05). In the Control sections, no significant differences were observed between the groups (*p* = 0.17). (Fig. 3.A)

**Table 2:** Post-hoc comparison p-values of Penetration percentage & Arc length Percentage

	Group	Compared to	*P* value
**Penetration % (test)**	**Manual Dynamic Agitation**	**Ultrasonic**	0.96
		**Diode Laser**	0.38
	**Ultrasonic**	**Diode Laser**	0.04*
**Arc length % (control)**	**Manual Dynamic Agitation**	**Ultrasonic**	0.13
		**Diode Laser**	0.04*
	**Ultrasonic**	**Diode Laser**	1.00

A table presenting the Bonferroni-adjusted *p*-values from the pairwise comparisons between activation techniques for parameters where the Kruskal-Wallis test indicated a significant difference. UAI demonstrated significantly superior Penetration Area (%) compared to LAI apical to the fracture (*p* = 0.04)

*Statistically significant at Bonferroni-adjusted significance level

### Maximum penetration depth

The mean penetration depth was highest in the MDA group (705.45 ± 233.04 μm), followed by UAI (630.29 ± 315.24 μm) and LAI (490.07 ± 242.16 μm) in the apical sections; however, this was not statistically significant (*p* = 0.14, Kruskal-Wallis test). No significant differences were observed between the groups in the control sections (*p* = 0.85). (Fig. 3.B)

### Arc length %

In the apical sections, the proportion of the canal circumference penetrated by the irrigant was comparable between all three techniques (*p* = 0.95), however, a significant difference was found in the control sections (*p* = 0.04). (Fig. 3.C)

### Comparison of apical & control sections

- For MDA and LAI, all three parameters were significantly reduced in the apical sections compared to the control sections (*p* ≤ 0.008 for all comparisons).
- For Ultrasonic Activation (UAI), a significant reduction in the apical sections was observed for MPD (*p* = 0.004) and Arc Length % (*p* = 0.003). Crucially, for Penetration Area %, UAI demonstrated no statistically significant difference between the Apical and Control sections (*p* = 0.10). (Table 1)

### Debris & smear layer scoring

The efficacy of the three activation techniques in removing the compacted debris and smear layer barrier apical to the fractured instrument was directly evaluated using scanning electron microscopy. The scores for smear layer and debris are presented in Table 3.

**Table 3:** Debris and smear layer scores for the three activation techniques

		Manual Dynamic Agitation	Ultrasonic Activation	Diode Laser Activation	*P* value
Smear layer	**Mean (SD)**	3.90 (0.85) **a**	2.40 (0.85) **b**	2.83 (0.92) **b**	< 0.001*
	**Median (IQR)**	4.00 (3.50, 4.50)	2.50 (2.00, 3.00)	3.00 (2.00, 3.50)	
Debris	**Mean (SD)**	3.60 (0.63) **a**	2.73 (0.90) **b**	2.93 (0.84) **b**	0.007*
	**Median (IQR)**	3.50 (3.50, 4.00)	3.00 (2.00, 3.50)	3.00 (2.50, 3.50)	

Data are presented as Mean (Standard Deviation) and Median (Interquartile Range). Different superscript letters (a, b) indicate statistically significant differences between groups based on post-hoc pairwise comparisons with Bonferroni adjustment. The p-value is from the Kruskal-Wallis H test (statistically significant *P* < 0.05)

The Kruskal-Wallis H test revealed statistically significant differences among the three activation techniques for both smear layer (*p* < 0.001) and debris (*p* = 0.007) scores.

Post-hoc pairwise comparisons with Bonferroni adjustment (Table 4) clarified these differences. For both smear layer and debris removal, Manual Dynamic Agitation (MDA) demonstrated significantly higher scores (indicating poorer cleaning) compared to both Ultrasonic Activation (UAI) (Smear: *p* < 0.001; Debris: *p* = 0.01) and Diode Laser Activation (LAI) (Smear: *p* = 0.01; Debris: *p* = 0.04).

**Table 4:** Post-hoc pairwise comparison *p*-values (Bonferroni-adjusted) for debris and smear layer scores

	Group	Compared to	*P* value
**Smear layer**	**Manual Dynamic Agitation**	**Ultrasonic Activation**	< 0.001*
		**Diode Laser Activation**	0.01*
	**Ultrasonic Activation**	**Diode Laser Activation**	0.76
**Debris**	**Manual Dynamic Agitation**	**Ultrasonic Activation**	0.01*
		**Diode Laser Activation**	0.04*
	**Ultrasonic Activation**	**Diode Laser Activation**	1.00

Statistically significant differences (*p* < 0.05) are highlighted in bold

**Fig. 4:**
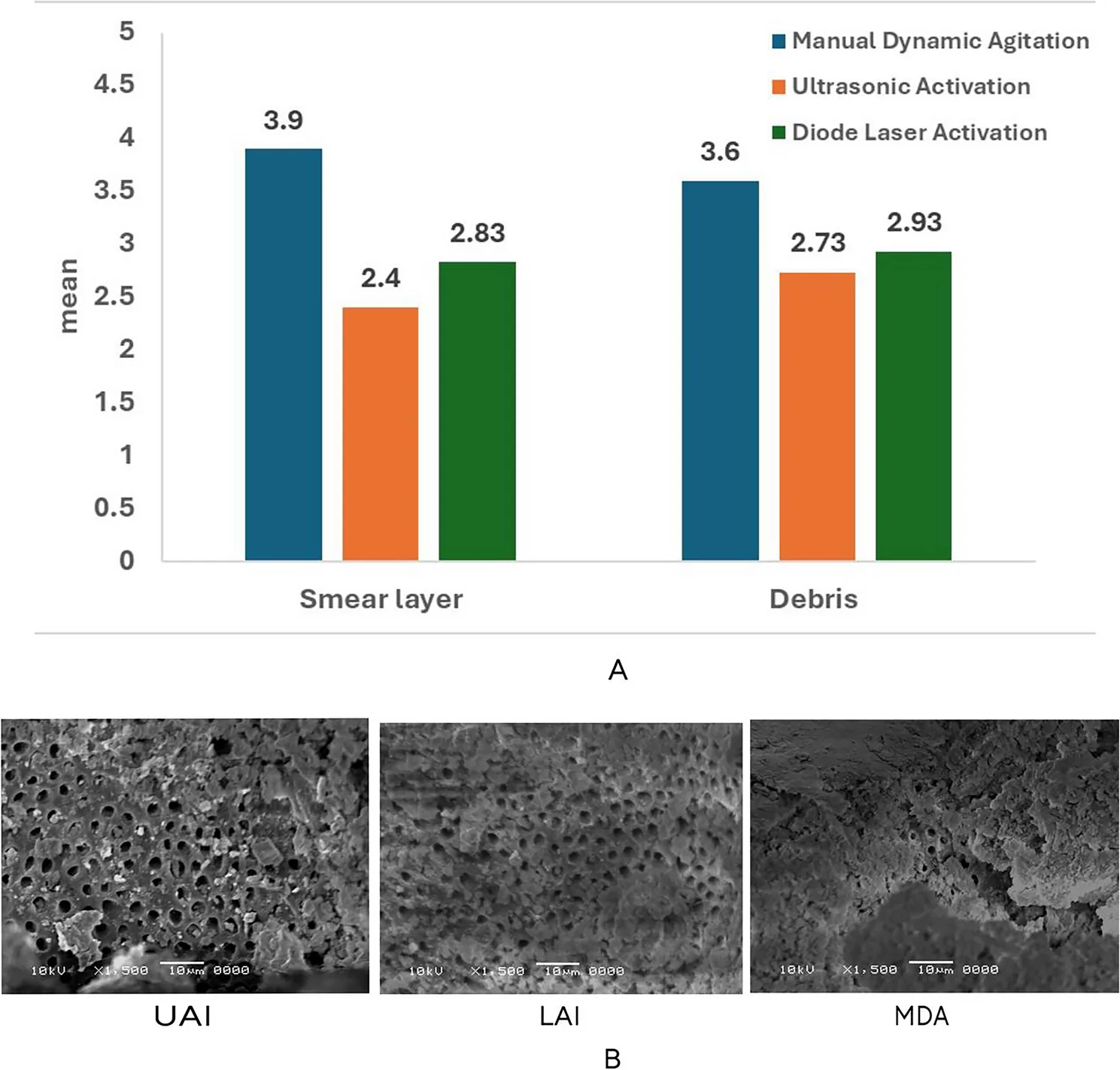
Graphical and representative scanning electron microscopy (SEM) evaluation of debris and smear layer removal. **A** Bar charts comparing the smear layer and debris mean scores for the three activation techniques: Manual Dynamic Agitation (MDA), Ultrasonic Activation (UAI), and Diode Laser Activation (LAI). Lower scores indicate better removal. **B** Representative SEM micrographs (magnification 1500x) of the root canal wall in the apical third for each group, provided as illustrative examples of the qualitative appearance corresponding to the quantitative scores in (**A**) smear layer bar chart

Critically, there was no statistically significant difference in the cleaning efficacy between Ultrasonic Activation and Diode Laser Activation for either smear layer (*p* = 0.76) or debris (*p* = 1.00) removal (Fig. 4).

## Discussion

The management of apically fractured instruments presents a significant clinical challenge, primarily due to the compacted dentinal debris and smear layer that forms a formidable barrier to clean around and apical to the fragment. This study employed a novel dual-method approach to evaluate the efficacy of three irrigation activation techniques in overcoming this barrier by simultaneously assessing irrigant penetration patterns (CLSM) and direct cleaning efficacy (SEM). The most significant finding is the superior performance of Ultrasonic Activation (UAI) in restoring irrigant penetration area, while UAI and Diode Laser Activation (LAI at 2.0 W) showed no significant difference in direct debris and smear layer removal.

The superior performance of UAI in overcoming the vapor lock barrier and restoring irrigant penetration, as measured by CLSM, aligns with a substantial body of evidence. A 2023 systematic review and meta-analysis by Raj et al. concluded that among various activation techniques, Passive Ultrasonic Irrigation (PUI), which employs the same fundamental mechanism as the Ultrasonic Activation (UAI) used in this study, was one of the most effective methods for delivering irrigant to the working length in both straight and curved canals, significantly outperforming manual dynamic agitation [20]. This review highlighted the role of acoustic streaming and cavitation in enhancing the efficacy of irrigants. Furthermore, the critical role of the vapor lock effect, which UAI successfully overcame in our model, has been well-documented. Tay et al. demonstrated that the presence of an apical vapor lock significantly adversely affects debridement efficacy in a closed canal system, creating a clinical scenario very similar to one with an apically fractured instrument [4]. Our findings directly build upon this, showing that UAI’s mechanism is powerful enough to disrupt this barrier. Another 2025 study comparing activation systems also found that PUI and LAI were the most effective for smear layer removal in the challenging apical third of curved canals, which is consistent with our SEM results [21].

Manual Dynamic Agitation (MDA) was chosen as the control group rather than conventional syringe irrigation (CSI) because CSI is well-established to be ineffective in overcoming the apical vapor lock in a closed-ended model [4]. Boutsioukis et al. (2014) [5] observed a vapor lock in 48% of cases during syringe irrigation. In contrast, MDA is an established activation technique that has been used as a comparator in multiple studies and consistently outperforms CSI for smear layer removal [22–24]. Furthermore, MDA represents the simplest clinically available manual technique and is superior to passive irrigation, as confirmed by a systematic review ranking irrigation techniques according to their ability to push the irrigant to the working length as: ANP > PUI > Sonic > MDA > CNI [20]. Thus, MDA served as a clinically meaningful baseline comparator against which the machine-assisted techniques (UAI and LAI) could be assessed.

A critical consideration when using ultrasonic activation near an apically separated instrument is the potential for iatrogenic displacement of the fragment. The application of ultrasonic energy, especially if not meticulously controlled, could theoretically lead to the fragment being pushed further apically. This concern is substantiated by expert consensus and clinical guides, which explicitly list apical extrusion of the file as a known risk during retrieval attempts [3]. Therefore, the non-contact irrigation activation protocol used in the present study, where the ultrasonic tip was placed 1 mm coronal to the fracture without direct contact, was designed specifically to leverage the benefits of acoustic streaming while mitigating the physical forces that could displace the fragment.

Our finding that Diode Laser Activation (at 2.0 W) showed no significant difference to UAI in debris and smear layer removal is a key result that requires careful contextualization. This contrasts with studies that used different laser parameters or types. For instance, some studies utilizing Er: YAG lasers for Laser-Activated Irrigation (LAI) have demonstrated very broad dispersion patterns and strong debridement, often attributed to powerful photoacoustic shockwaves [21]. Our results, using a 970 nm diode laser, demonstrate that a clinically safe, mid-power protocol for this specific laser type can achieve cleaning efficacy that is not statistically different than ultrasonics. However, it is crucial to acknowledge that other research has ranked the efficacy of activation techniques differently. The mentioned 2023 meta-analysis found Apical Negative Pressure (ANP) to be the most effective technique overall, followed by PUI, sonic irrigation, and then MDA, suggesting that the optimal technique can vary based on the desired outcome and model used [20]. Furthermore, a study on calcium hydroxide removal found that a sonic device (EndoActivator) performed best, followed by PUI, and then MDA, indicating that for some tasks, sonic energy can rival or exceed ultrasonic performance [25]. This discrepancy highlights that efficacy can be task dependent.

The 2.0 W continuous-wave setting employed in this study has been shown to be thermally safe for the periodontium using the same 970 nm wavelength, with temperature rises remaining below the critical 10 °C threshold (Tanner et al., 2024; Saban et al., 2025) [26–28]. Furthermore, Pradhan and Karnik (2017) [29] evaluated a 940 nm diode laser with a 2.0 W continuous-wave mode and confirmed that these parameters can be safely used as an irrigation activation method. The fibre tip was positioned 1 mm coronal to the fractured fragment without contacting the metal, and continuous irrigation acted as a heat sink, minimizing any theoretical laser-metal interaction. The 10-second interval between cycles provides adequate cooling, consistent with published protocols [30].

A key strength of this study was the use of both CLSM and SEM, which revealed that while UAI and LAI were equally effective at cleaning the canal wall (SEM), UAI was superior in restoring the overall irrigant penetration area (CLSM). This suggests that while LAI’s photothermal convection is effective at agitating fluid immediately adjacent to the canal wall, the acoustic streaming of UAI may create a broader, more uniform fluid exchange in the entire root canal space beyond the fracture. This nuanced finding would have been missed with a single evaluation method and underscores the importance of multi-faceted assessment. The underperformance of MDA across both evaluation methods confirms its limitations in generating the intense hydrodynamic forces required to overcome a significant physical barrier, a finding consistent with the broader literature that places MDA as less effective than machine-assisted systems [20, 21].

The use of a 200 μm intracanal fiber placed 1 mm coronal to the obstruction was designed to maximize fluid agitation in the confined apical space, a more direct approach than coronal “pool” activation [31]. This study employed a 970 nm diode laser, a wavelength known for its strong absorption by water, to ensure effective photothermal agitation of the irrigant [32].

This in vitro study has inherent limitations; It is acknowledged that the standardized, non-bypassable fracture model employed in this study, while providing essential experimental control, cannot fully replicate the clinical heterogeneity of real fracture scenarios, including variations in fragment length, canal anatomy, curvature, and operator variability. This inherent limitation restricts the external validity of our findings, and clinical translation should consider these factors.

It is also acknowledged that Rhodamine B’s affinity for dentin collagen may overestimate true sealer penetration [33]. This phenomenon is a recognized constraint in studies utilizing fluorescent dyes for quantitative penetration analysis [33, 34]. However, the consistent application of the protocol across all experimental groups—including the use of a standardized RhB concentration, a mandatory 24-hour distilled water immersion to dissolve unbound dye [35], and identical CLSM imaging parameters—ensures that any potential overestimation affects all groups uniformly. Therefore, while absolute penetration values may be influenced by dye-specific artifacts, the relative differences observed between the activation techniques remain valid and quantitatively meaningful.

Another CLSM limitation is the potential interaction between Rhodamine B and sodium hypochlorite, which can affect fluorescence intensity [36]. However, the Rhodamine B–NaOCl mixture has been widely employed in endodontic CLSM studies evaluating irrigant penetration into dentinal tubules [37–39]. Notably, our 60-second activation period is substantially shorter than the exposure times investigated in the Tiong and Price (2012) [36] study, where significant dye degradation was observed under prolonged ultrasonic irradiation. Importantly, the same dye–NaOCl mixture and identical rinsing protocols were applied uniformly across all groups in the present study. Thus, any potential fluorescence attenuation affects all groups equally, and the relative differences observed between activation techniques remain valid.

Furthermore, the fractured instrument segment was removed prior to SEM evaluation to permit unobstructed visualisation of the canal wall. The authors acknowledge that this methodological requirement may not fully replicate the clinical scenario and could potentially overestimate the cleaning efficacy observed, as the physical presence of the fragment was eliminated before imaging. However, this step was necessary for the unobstructed visualization of the root canal wall and was applied uniformly across all experimental groups, ensuring that comparisons between activation techniques remain valid.

The relatively modest sample size within each experimental group may limit the precision and generalizability of the findings. Future studies with larger sample sizes are needed to confirm these results. Finally, this study focused on hydrodynamic efficacy and mechanical debridement; the subsequent biological outcome on biofilm elimination was not assessed.

The present findings offer several considerations for clinical practice. In the scenario of an apically fractured instrument managed conservatively, the use of machine-assisted activation (UAI or LAI) appears to be more effective than manual dynamic agitation for debris and smear layer removal, based on the present in vitro data. Ultrasonic activation demonstrated greater apical penetration area compared to diode laser activation, which may be relevant when enhanced fluid distribution in the apical region is desired. Diode laser activation showed no statistically significant difference from ultrasonic activation for debris and smear layer removal and, being a non-contact technique, may theoretically reduce the risk of fragment displacement. However, it must be emphasised that these observations are derived from an ex-vivo model and do not constitute definitive clinical evidence. The choice between UAI and LAI should be made cautiously, considering equipment availability, operator experience, and individual case characteristics. Further clinical studies are necessary to validate these findings and establish their relevance to patient outcomes.

Future research should build upon these findings by assessing the efficacy of different activation techniques against established biofilm models in the presence of an apical fragment. Evaluating bacterial viability using methods such as confocal microscopy or colony-forming unit counts under SEM would provide critical data on true disinfection potential.

## Conclusion

Within the limitations of this in-vitro model, ultrasonic activation produced greater apical penetration area than diode laser activation, while ultrasonic and diode laser activation produced significantly better debris and smear-layer removal than manual dynamic agitation. No statistical difference was observed between ultrasonic and diode laser activation for SEM-based cleaning scores. Further studies incorporating thermal safety assessment and biofilm models are required before definite clinical recommendations can be made.

## Data Availability

The datasets used and/or analyzed during the current study are available from the corresponding author on reasonable request.

## Abbreviations

ANP: Apical Negative Pressure
CLSM: Confocal Laser Scanning Microscopy
CNI: Conventional Needle Irrigation
EDTA: Ethylenediaminetetraacetic acid
IQR: Interquartile Range
LAI: Laser-Activated Irrigation (Diode Laser Activation)
MDA: Manual Dynamic Agitation
MPD: Maximum Penetration Depth
NaOCl: Sodium Hypochlorite
NiTi: Nickel-Titanium
PBS: Phosphate-Buffered Saline
PUI: Passive Ultrasonic Irrigation (used interchangeably with UAI in the literature to describe the same non-contact ultrasonic activation technique)
RPM: Revolutions Per Minute
SD: Standard Deviation
SEM: Scanning Electron Microscopy
UAI: Ultrasonic Activation
WL: Working Length
µm: Micrometer
